# Association between C10X polymorphism in the *CARD8* gene and inflammatory markers in young healthy individuals in the LBA study

**DOI:** 10.1186/s12872-024-03765-7

**Published:** 2024-02-13

**Authors:** Karin Fransén, Ayako Hiyoshi, Geena V. Paramel, Anita Hurtig-Wennlöf

**Affiliations:** 1 Cardiovascular Research Centre, School of Medical Sciences, Faculty of Medicine and Health, Örebro University, Örebro, Sweden; 2 Clinical Epidemiology and Biostatistics, School of Medical Sciences, Örebro University, Örebro, Sweden; 3 Department of Clinical Diagnostics, School of Health and Welfare, Jönköping University, Jönköping, Sweden

**Keywords:** Inflammation, CARD8, IL-6, Polymorphism, CCL20

## Abstract

**Background:**

The Caspase activation and recruitment domain 8 (CARD8) protein is a component of innate immunity as a negative regulator of NF- ĸB, and has been associated with regulation of proteins involved in inflammation. Expression of *CARD8* mRNA and protein has been identified in human atherosclerotic lesions, and the truncated T30A variant (rs2043211) of *CARD8* has been associated with lower C-reactive (CRP) and MCP-1 levels in myocardial infarction patients. The present study examines the role of a genetic variation in the *CARD8* gene in relation to a selection of markers of inflammation.

**Methods:**

In a cross-sectional study of young healthy individuals (18.0–25.9 yrs, *n* = 744) the association between the rs2043211 variant in the *CARD8* gene and protein markers of inflammation was assessed. Genotyping of the *CARD8* C10X (rs2043211) polymorphism was performed with TaqMan real time PCR on DNA from blood samples. Protein levels were studied via Olink inflammation panel (https://olink.com/). Using linear models, we analyzed men and two groups of women with and without estrogen containing contraceptives separately, due to previous findings indicating differences between estrogen users and non-estrogen using women. Genotypes were analyzed by additive, recessive and dominant models.

**Results:**

The minor (A) allele of the rs2043211 polymorphism in the *CARD8* gene was associated with lower levels of CCL20 and IL-6 in men (CCL20, Additive model: *p* = 0.023; Dominant model: *p* = 0.016. IL-6, Additive model: *p* = 0.042; Dominant model: *p* = 0.039). The associations remained significant also after adjustment for age and potential intermediate variables.

**Conclusions:**

Our data indicate that CARD8 may be involved in the regulation of CCL20 and IL-6 in men. No such association was observed in women.

These findings strengthen and support previous in vitro data on IL-6 and CCL20 and highlight the importance of *CARD8* as a factor in the regulation of inflammatory proteins. The reason to the difference between sexes is however not clear, and the influence of estrogen as a possible factor important for the inflammatory response needs to be further explored.

**Supplementary Information:**

The online version contains supplementary material available at 10.1186/s12872-024-03765-7.

## Background

Cardiovascular diseases (CVDs), such as coronary artery disease (CAD) and cerebrovascular disease are mainly a result of atherosclerosis, which is a slowly progressing chronic inflammatory disease that results in narrowing of large and medium-sized arteries. The atherosclerotic process starts in early life as fatty streaks, which typically evolves asymptomatically over decades, followed by accumulation of inflammatory cells, apoptotic and necrotic cells, debris and cholesterol in the plaque [1]. Among childhood risk factors for CVD are childhood adiposity, hypertension and hyperlipidemia [2]. Inflammation is a central process in the pathogenesis of atherosclerosis and recently we identified CARD8 (also known as TUCAN/CARDINAL/NDDP1) as a factor important for regulation of inflammatory proteins in endothelial cells [3]. The CARD8 protein is a component of the innate immunity and was in an early study identified as a negative regulator of NF-κB via its interaction with the regulatory subunit of the IĸB kinase complex [4]. Furthermore, CARD8 physically binds and regulates procaspase-9 and caspase-1 and regulates generation of IL-1β via caspase-1 [5, 6]. Due to this, CARD8 has been suggested as a part of the NLRP3 inflammasome, although its role in IL-1β release has been debated. The CARD8 protein has also been shown to negatively regulate NLRP3 and IL-1β secretion in vitro in HEK293 cells [7] but in vascular smooth muscle cells, CARD8 did not have any impact on the IL-1β release, possibly indicating on a cell-specific regulation [8].

Knowledge about the role of CARD8 in general is still limited, although research on CARD8 is increasing. Recently, CARD8 was identified as an inflammasome sensor and studies have shown that CARD8 binds dipeptidyl peptidases (DPP)8 and 9 [9–11]. Furthermore, CARD8 is degraded by the core 20S proteasome and controls the activation of the CARD8 inflammasome [12]. The knowledge on the regulatory function of CARD8 in the etiology of CVDs is also limited. In two of our previous studies, we showed upregulation of *CARD8* mRNA and protein expression in human atherosclerotic lesions compared to non-atherosclerotic vessels, indicating a pathophysiological role of CARD8 in CVD [3, 13]. On the other hand, the role of genetic alterations in the *CARD8* gene has been extensively investigated for the association to various inflammatory diseases, including CVDs [14]. The T30A polymorphism (rs2043211) in exon 5 encoding a C10X alteration of the *CARD8* gene and has been extensively studied and causes a premature stop codon that terminates the protein, although the functional aspect of the polymorphism is not completely clear. In one of our previous studies, we have shown an association between lower mRNA expression of *CARD8* in atherosclerotic lesions from homozygous polymorphic individuals for the C10X polymorphism rs2043211, where the minor allele was associated with lower C-reactive protein (CRP) and MCP-1 levels [13]. We have also identified that CARD8 acts as a regulator of inflammatory cytokines and chemokines like CXCL1, CXCL6, PDGF-A, MCP-1 and IL-6 in endothelial cells and atherosclerotic lesions [3], which indicates that CARD8 plays an important role for the regulation of proteins involved in inflammation. Knowledge about the role of the rs2043211 *CARD8* variant on the expression of proteins involved in inflammation in young healthy adults is however limited.

The aim of the present study was to examine the role of the rs2043211 C10X polymorphism in the *CARD8* gene in relation to a selection of markers of inflammation previously shown altered after *CARD8* knock down [3]. We have hypothesized that the minor variant of the C10X polymorphism mimics the lack of CARD8 protein.

## Methods

### Study population, ethics and exclusion criteria

Blood samples from 834 Swedish young self-reported non-smoking healthy individuals (aged 18.0–25.9 years) were collected in the Lifestyle, Biomarkers and Atherosclerosis (LBA) cohort, Örebro University, Sweden.

The LBA study is an epidemiological cross-sectional study on cardiovascular risk factors, including biomarkers, vascular function variables and physical activity assessment. Details about the LBA cohort and sampling were published in Fernström et al. [15]. Informed consent was obtained from all participants. The study was approved by Regional Ethical Review Board, Uppsala, Sweden (Dnr 2014/224) and performed according to the Helsinki declaration.

**Fig. 1:**
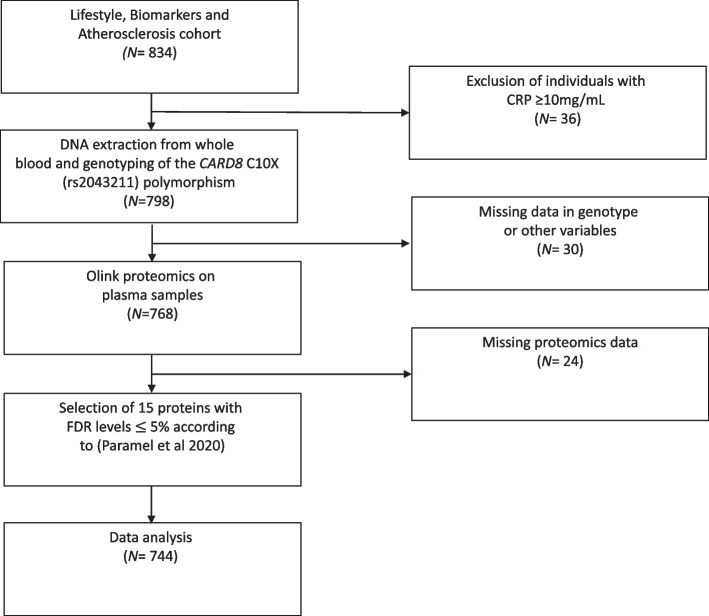
Flow chart of the present study and selection of study specimens in the Lifestyle, Biomarkers and Atherosclerosis cohort

Out of 834 individuals, we excluded individuals whose CRP level was ≥ 10mg/mL (*n* = 36), indicating ongoing inflammation, and had missing data in genotype (*n* = 15; failed quality control) and/or other variables (*n* = 15). Further 24 individuals were excluded due to missing data in proteins. In total, we therefore included the remaining *n* = 744 individuals in the present study, whereof 511 were females and 233 males (Fig. 1).

### DNA extraction and genotyping of the *CARD8* C10X (rs2043211) polymorphism

Genomic DNA was isolated with Wizard Genomic DNA purification kit (Promega, Madison, WI) according to the supplier’s recommendations. DNA concentration was measured with NanoDrop 200 spectrophotometer (Thermo Fisher Scientific, Waltham, MA) and diluted to 20 ng/µl.

Genotyping of the rs2043211 polymorphism in the *CARD8* gene was performed for the LBA cohort. Ten nanograms of DNA was amplified in a 10 µl reaction containing 1 × TaqMan Genotyping Mastermix (Applied Biosystems, Foster City, CA) and 1 × TaqMan SNP Genotyping Assay (Applied Biosystems) with predesigned primers and probes C_ _11708080_1_ (Applied Biosystems) according to a TaqMan standard protocol in a QuantStudio 7 Flex Real-Time PCR system (Applied Biosystems) followed by allelic discrimination analysis. Four reactions in each 96-well plate contained no-template control. A random selection of > 10% of all samples were re-genotyped in a new PCR reaction to verify the accuracy of the genotyping. The genotypes were analyzed by three classifications: additive, recessive and dominant models. Additive model assigned values of 0, 0.5 and 1 for the homozygous wildtype (TT), heterozygous (TA) and polymorphic homozygous (AA) genotype groups, respectively. The dominant model compared TA + AA with TT (reference) and the recessive model compared AA *vs*. TT + TA (reference).

### Olink proteomics

Proteomics analysis was performed with Olink (www.olink.com) proteomics of plasma samples from the LBA cohort as described in Pettersson Pablo et al. [16, 17].

Based on the previous results by Paramel et al. [3], we selected the 16 proteins from the Olink Inflammation and CVDIII panels (http://olink.com) with false discovery rate (FDR) levels $\le$ 5%: CXCL6, CCL20, IL-6, IL-18R1, CXCL-1, ADA, CD40, 4E-BP1, MCP3, MCP1, IL-8, TWEAK, VEGF-A, OPG, t-PA and AXL. Of these, MCP3 was not included in the present study because more than 50% of the individuals had protein levels under the Limit of Detection (LOD) value, resulting in 15 proteins analyzed. The values of the proteins were log2 transformed to fit normal distribution.

Five individuals had missing values in these protein data, and we replaced these missing data with the mean of observed values. A protein value equal to or greater (or smaller) than 4 standard deviations from the mean were replaced to missing values. The number of such observations differed by protein, but it ranged between 0 (7 proteins) and 5 (CCL20).

### Other variables

The variables age, Body Mass Index (BMI; kg/m2), Low density lipoprotein (LDL; mmol/L), triglycerides (mmol/L), insulin (mU/L), and systolic blood pressure (mm Hg) have previously been suggested to be involved in inflammatory processes [18, 19]. BMI, LDL, triglycerides, insulin, and systolic blood pressure were measured according to standard procedures as previously described in Fernström et al. [15].

Further, we examined whether men or women, with the latter being further separated into estrogen-containing contraceptive usage (yes/no) modifies the association between genotyping of the rs2043211 polymorphism and proteins. Among women, estrogen-containing contraceptive usage was self-reported in a questionnaire. Hereafter, women using estrogen containing contraceptive are called “estrogen users (EU)”, and women not using them as “non-estrogen users (NEU)”. Details of the reported estrogen data are described in Pettersson-Pablo et al. [20].

We assumed that the rs2043211 could also be involved in the regulation of inflammatory proteins in a similar pattern as previously described [18, 19]. Therefore, we adjusted for these variables to obtain the extent to which the rs2043211 polymorphism is associated with protein expression independently.

### Statistics

The characteristics of the participants were summarized by frequencies, proportions, means and standard deviations (or median and 25 and 75 percentiles for skewed data). Box plots were used to summarize the distribution of protein values by TT, TA and AA separately for men and NEU and EU women. The associations between additive, dominant or recessive models with the fifteen proteins were assessed using linear models for microarray data (using the R package ‘*limma’*, [21, 22]). It fits a linear model to each protein while analyzing the entire experiment as an integrated whole to increase effectiveness in estimation. For each of additive, dominant, and recessive models, we fitted two models: age-adjusted (Model 1) and a model adjusting for age, BMI, LDL, triglycerides, insulin, and systolic blood pressure (Model 2). All adjustment variables were modeled as piecewise linear functions using linear splines, which model the associations between these variables and proteins using three equally distanced segments. Two segments were used if three segments did not fit data. The estimates from Model 2 are interpreted as the association of the rs2043211 polymorphism with a given protein not mediated through variables adjusted for in the model. Two-sided p-values lower than 0.05 were considered statistically significant. Analytical data were prepared by Stata MP/17 and analyses were conducted using RStudio version 1.2.5033.

## Results

### Genotype distribution in the *CARD8* gene

The allele frequencies of the rs2043211 polymorphism were 69% for the major (T) allele and 31% for the minor (A) allele (Table 1). The rs2043211 polymorphism in the *CARD8* gene was found in Hardy Weinberg equilibrium.

**Table 1:** Distribution of the genotypes in the rs2043211 polymorphism in the *CARD8* gene based on gender and estrogen use in the Lifestyle, Biomarkers and Atherosclerosis (LBA) cohort. The genotypes were displayed in additive (TT *vs*. TA *vs*. AA), dominant (TA + AA *vs*. TT) and recessive models (AA *vs*. TT + TA)

***Additive model***				
	**Total**	**TT**	**TA**	**AA**
	***n***	***n*** ** (%)**	***n*** ** (%)**	***n*** ** (%)**
Men	233	101 (43)	105 (45)	27 (12)
NEU women	383	176 (46)	174 (45)	33 (9)
EU women	128	69 (54)	51 (40)	8 (6)
**Total**	744	346 (47)	330 (44)	68 (9)
***Dominant model***				
	**Total**	**TT**		**TA + AA**
	***n***	***n*** ** (%)**		***n*** ** (%)**
Men	233	101 (43)		132 (57)
NEU women	383	176 (46)		207 (54)
EU women	128	69 (54)		59 (46)
**Total**	744	346 (47)		398 (53)
***Recessive model***				
	**Total**	**TT + TA**		**AA**
	***n***	***n*** ** (%)**		***n*** ** (%)**
Men	233	206 (88)		27 (12)
NEU women	383	350 (91)		33 (9)
EU women	128	120 (94)		8 (6)
**Total**	744	676 (91)		68 (9)

### Basic characteristics of the LBA cohort based on genotypes in the rs2043211 polymorphism in the *CARD8* gene

The distribution of genotype TT, TA or AA for 744 individuals was stratified by men, NEU and EU women (Table 1). The distribution of age and biomarker characteristics are shown in Table 2. The results were presented as mean or median, and standard deviation (SD) or quartiles respectively, depending on the distribution of the variables (insulin and CRP were skewed).

**Table 2:** Basic characteristics of the Lifestyle, Biomarkers and Atherosclerosis (LBA) cohort based on genotypes in the rs2043211 polymorphism in the *CARD8* gene

	**Total**	**TT**	**TA**	**AA**
**Men (** ***n*** ** = 233)** ^**a**^				
Age (years)	22.0 (2.0)	21.9 (2.0)	22.0 (2.1)	22.8 (1.6)
Height (cm)	181.7 (6.7)	181.6 (6.3)	181.5 (7.1)	182.4 (7.0)
Weight (kg)	77.7 (11.5)	77.0 (10.7)	78.1 (12.8)	79.2 (9.0)
BMI (kg/m^2^)	23.5 (3.1)	23.3 (3.0)	23.7 (3.4)	23.8 (2.4)
LDL (mmol/L)	2.3 (0.7)	2.2 (0.6)	2.4 (0.8)	2.5 (0.6)
Triglycerides (mmol/L)	0.8 (0.4)	0.8 (0.3)	0.8 (0.4)	0.9 (0.3)
Systolic blood pressure (mm Hg)	124.9 (11.9)	123.9 (12.5)	126.2 (11.9)	123.5 (9.5)
Insulin (mU/L)	6.8 (4.9, 9.1)	6.8 (5.0, 9.4)	7.0 (4.9, 9.1)	6.7 (4.3, 8.8)
CRP (mg/L)	0.5 (0.3, 1.1)	0.4 (0.3, 1.0)	0.6 (0.2, 1.5)	0.6 (0.2, 1.0)
**Non-estrogen using women (** ***n*** ** = 383)** ^**a**^				
Age (years)	21.9 (2.0)	22.1 (1.9)	21.7 (2.0)	21.8 (2.0)
Height (cm)	168.6 (6.6)	168.3 (6.1)	168.9 (7.1)	168.8 (6.3)
Weight (kg)	63.7 (11.1)	64.2 (10.5)	63.1 (11.8)	63.9 (11.0)
BMI (kg/m^2^)	22.4 (3.5)	22.6 (3.4)	22.1 (3.5)	22.4 (3.7)
LDL (mmol/L)	2.2 (0.6)	2.2 (0.7)	2.2 (0.6)	2.2 (0.6)
Triglycerides (mmol/L)	0.7 (0.3)	0.7 (0.3)	0.7 (0.3)	0.9 (0.5)
Systolic blood pressure (mm Hg)	109.8 (8.8)	110.3 (9.1)	109.2 (8.2)	110.5 (10.1)
Insulin (mU/L)	7.0 (5.1, 9.8)	7.0 (5.1, 9.7)	6.8 (5.1, 9.6)	8.4 (6.7, 10.5)
CRP (mg/L)	0.6 (0.3, 1.1)	0.5 (0.3, 1.1)	0.5 (0.3, 1.0)	0.7 (0.4, 1.1)
**Estrogen-using women (** ***n*** ** = 128)** ^**a**^				
Age (years)	21.7 (1.8)	21.7 (1.8)	21.8 (1.9)	21.6 (1.4)
Height (cm)	168.6 (5.8)	168.0 (6.2)	169.5 (5.3)	168.0 (5.6)
Weight (kg)	63.2 (10.6)	62.3 (8.5)	64.8 (13.3)	60.3 (5.5)
BMI (kg/m^2^)	22.2 (3.1)	22.0 (2.5)	22.5 (3.9)	21.4 (1.8)
LDL (mmol/L)	2.6 (0.8)	2.6 (0.9)	2.5 (0.7)	3.1 (0.9)
Triglycerides (mmol/L)	1.0 (0.4)	1.0 (0.4)	1.0 (0.4)	1.0 (0.6)
Systolic blood pressure (mm Hg)	112.8 (8.9)	112.1 (8.6)	114.2 (9.1)	109.6 (9.8)
Insulin (mU/L)	7.1 (4.9, 10.1)	6.8 (4.4, 10.1)	7.0 (5.6, 9.9)	9.0 (7.5, 10.3)
CRP (mg/L)	1.6 (0.7, 3.1)	1.9 (0.9, 3.1)	1.0 (0.6, 3.0)	2.3 (0.5, 3.9)

^a^Age, height, weight, BMI, LDL, triglycerides and systolic blood pressure were presented as Mean and Standard deviation (SD). Insulin and CRP were presented as Median and 25 and 75 percentiles

The distribution of covariates such as BMI, blood pressure, LDL, insulin, and CRP were within the age and gender specific expected values, and there appeared to be little differences by genotypes. Distribution of the protein levels by TT, TA or AA for men, NEU and EU women is available in Supplementary material S1.

### Association between rs2043211 genotype in the *CARD8* gene and inflammatory proteins in the LBA cohort

The *CARD8* polymorphism rs2043211 generates a truncated variant in codon 10 of the *CARD8* gene (C10X). We evaluated the association between 15 proteins assessed via Olink proteomics and the *CARD8* genotype, previously found to be regulated by CARD8 [3]. In the present cohort of healthy, young adults, we assumed a similar effect of the minor (A) allele (encoding the truncated X variant) of the polymorphism rs2043211 as the effect of *CARD8* knock-down in HUVECs [3]. The script used is available in Supplementary material S2.

### Additive model

In men, the minor (A) allele of the rs2043211 polymorphism in the *CARD8* gene was significantly associated with lower levels of CCL20 (*p* = 0.023, Model 1; Table 3) and IL-6 (*p* = 0.042, Model 1; Table 3) in the age adjusted estimate. The association remained after adjustment for BMI, LDL and other potential intermediating variables (CCL20: *p* = 0.030; IL-6: *p* = 0.039, Model 2; Table 3). There were no such relations found in NEU or EU women.

**Table 3:** Association according to the additive model (TT vs. TA vs. AA), between the rs2043211 polymorphism in the CARD8 gene encoding C10X truncated variant and expression of 15 proteins studied by Olink proteomics. A value of 0 was assigned to TT, 0.5 to TA, and 1 to AA

	Men					NEU women					EU Women				
	Model 1^a^			Model 2^b^		Model 1^a^			Model 2^b^		Model 1^a^			Model 2^b^	
	Coefficient	*p*		Coefficient	*p*	Coefficient	*p*		Coefficient	*p*	Coefficient	*p*		Coefficient	*p*
ADA	0.008	0.926		0.005	0.956	-0.017	0.793		-0.012	0.856	0.047	0.696		0.038	0.760
AXL	0.105	0.118		0.106	0.117	-0.091	0.069		-0.085	0.095	-0.004	0.968		0.004	0.969
CCL20	-0.361	0.023		-0.353	0.030	0.128	0.311		0.080	0.538	0.174	0.451		0.162	0.495
CD40	-0.177	0.027		-0.139	0.084	-0.080	0.271		-0.098	0.183	0.088	0.510		0.102	0.464
CXCL1	-0.152	0.296		-0.042	0.773	0.117	0.271		0.113	0.300	0.362	0.064		0.420	0.033
CXCL6	-0.200	0.177		-0.133	0.368	0.047	0.723		0.030	0.822	0.421	0.134		0.361	0.218
IL-6	-0.238	0.042		-0.241	0.039	0.111	0.300		0.092	0.358	0.020	0.923		0.005	0.981
IL-8	-0.015	0.878		0.006	0.951	0.059	0.448		0.030	0.705	0.003	0.986		-0.025	0.892
IL-18R1	0.063	0.404		0.049	0.513	0.066	0.302		0.049	0.429	0.052	0.644		0.053	0.630
MCP-1	0.087	0.151		0.108	0.082	0.007	0.887		0.003	0.956	-0.032	0.753		-0.026	0.811
OPG	0.113	0.034		0.109	0.049	0.013	0.788		-0.003	0.954	0.006	0.956		0.005	0.961
t-PA	0.007	0.974		0.061	0.785	0.149	0.463		0.000	1.000	0.037	0.910		-0.022	0.944
TWEAK	-0.069	0.225		-0.071	0.236	0.010	0.860		-0.012	0.833	0.185	0.147		0.158	0.222
VEGF-A	-0.026	0.698		-0.024	0.706	-0.025	0.684		-0.026	0.670	-0.082	0.464		-0.084	0.472
4E-BP1	-0.065	0.617		-0.083	0.520	-0.025	0.825		-0.036	0.759	0.186	0.390		0.156	0.493

^a^Model 1 is adjusted for age

^b^Model 2 model is adjusted for age, height, weight, body mass index, low density lipoprotein, triglycerides, systolic blood pressure, insulin, and C-reactive protein

A few of the statistically significant observations in this study were not consistent with previous findings: In men and EU women respectively, the minor allele was significantly associated with a higher value in OPG and CXCL1 (Table 3), but the opposite direction as previously reported [3].

### Dominant model

In the dominant model in men, where TA + AA were grouped together and compared to TT (reference), the minor allele of *CARD8* was significantly associated with lower levels of CCL20 (*p* = 0.016, Model 1; *p* = 0.030, Model 2; Table 4) and IL-6 (*p* = 0.039, Model 1; *p* = 0.045, Model 2; Table 4). The OPG level showed positive association with the minor allele in Model 1, but the association was slightly weakened after the adjustment (Table 4). The direction of associations in other proteins were inconsistent between men and NEU or EU women.

**Table 4:** Association according to the dominant model (TA + AA vs. TT as reference) between the rs2043211 polymorphism in the CARD8 gene encoding C10X truncated variant and expression of 15 proteins studied by Olink proteomics

	Men					NEU women					EU women				
	Model 1^a^			Model 2^b^		Model 1^a^			Model 2^b^		Model 1^a^			Model 2^b^	
	Coefficient	*p*		Coefficient	*p*	Coefficient	*p*		Coefficient	*p*	Coefficient	*p*		Coefficient	*p*
ADA	0.024	0.663		0.022	0.700	-0.032	0.457		-0.022	0.616	0.024	0.737		0.026	0.725
AXL	0.032	0.489		0.046	0.318	-0.052	0.113		-0.048	0.142	0.044	0.494		0.029	0.639
CCL20	-0.263	0.016		-0.241	0.030	0.051	0.525		0.027	0.744	0.142	0.300		0.136	0.334
CD40	-0.096	0.077		-0.072	0.187	-0.062	0.182		-0.062	0.191	0.059	0.452		0.080	0.340
CXCL1	-0.058	0.553		0.025	0.802	0.048	0.481		0.053	0.451	0.183	0.116		0.252	0.033
CXCL6	-0.141	0.157		-0.083	0.406	-0.021	0.806		-0.016	0.851	0.312	0.060		0.292	0.093
IL-6	-0.163	0.039		-0.159	0.045	0.019	0.788		0.024	0.713	-0.060	0.625		-0.089	0.440
IL-8	0.003	0.966		0.026	0.704	0.018	0.719		-0.001	0.984	0.021	0.837		0.008	0.938
IL-18R1	0.044	0.382		0.047	0.351	0.007	0.874		0.010	0.796	0.035	0.606		0.021	0.751
MCP-1	0.016	0.693		0.037	0.374	-0.005	0.893		-0.007	0.840	-0.011	0.864		-0.012	0.857
OPG	0.074	0.040		0.072	0.054	-0.001	0.970		-0.010	0.756	0.009	0.895		0.010	0.885
t-PA	-0.093	0.535		-0.030	0.840	0.074	0.570		0.022	0.862	0.033	0.869		0.034	0.861
TWEAK	-0.046	0.231		-0.047	0.248	0.006	0.865		-0.008	0.817	0.113	0.135		0.110	0.152
VEGF-A	-0.004	0.929		0.004	0.927	-0.022	0.580		-0.011	0.776	-0.068	0.304		-0.082	0.239
4E-BP1	-0.007	0.937		-0.013	0.884	-0.054	0.458		-0.047	0.532	0.069	0.590		0.062	0.648

^a^Model 1 is adjusted for age

^b^Model 2 model is adjusted for age, height, weight, body mass index, low density lipoprotein, triglycerides, systolic blood pressure, insulin and C-reactive protein

### Recessive model

In the recessive model, MCP-1 was positively associated with the minor allele of *CARD8* in the Models 1 and 2 in men, and the IL-18R1 was positively associated with the minor allele of *CARD8* in the Model 1 but not Model 2 in NEU women (Table 5). Both observations were in the opposite direction as compared to our previous study (Table 5; [3]). Further, AXL was positively associated with the minor allele of *CARD8* in men in Model 1, but not in Model 2, which was in the same direction as in Paramel et al. [3].

**Table 5:** Association according to the recessive model (AA with TA + TT as reference) between the rs2043211 polymorphism in the CARD8 gene encoding C10X truncated variant and expression of 15 proteins studied by Olink proteomics

	Men					NEU women					EU women				
	Model 1^a^			Model 2^b^		Model 1^a^			Model 2^b^		Model 1^a^			Model 2^b^	
	Coefficient	*p*		Coefficient	*p*	Coefficient	*p*		Coefficient	*p*	Coefficient	*p*		Coefficient	*p*
ADA	-0.041	0.632		-0.042	0.636	0.056	0.456		0.040	0.612	0.049	0.740		0.010	0.951
AXL	0.157	0.027		0.124	0.081	-0.071	0.216		-0.066	0.251	-0.201	0.127		-0.149	0.252
CCL20	-0.166	0.333		-0.205	0.234	0.170	0.227		0.122	0.407	-0.063	0.823		-0.098	0.740
CD40	-0.160	0.058		-0.134	0.112	-0.009	0.917		-0.052	0.535	0.024	0.882		0.014	0.938
CXCL1	-0.195	0.201		-0.151	0.326	0.149	0.213		0.121	0.333	0.387	0.108		0.344	0.162
CXCL6	-0.101	0.519		-0.094	0.544	0.187	0.207		0.131	0.392	-0.010	0.977		-0.092	0.802
IL-6	-0.133	0.283		-0.153	0.213	0.229	0.058		0.163	0.157	0.346	0.172		0.364	0.130
IL-8	-0.039	0.707		-0.047	0.655	0.096	0.279		0.080	0.370	-0.099	0.636		-0.139	0.535
IL-18R1	0.032	0.689		-0.005	0.953	0.149	0.037		0.093	0.186	0.018	0.899		0.015	0.913
MCP-1	0.154	0.016		0.147	0.023	0.033	0.580		0.029	0.631	-0.058	0.656		-0.044	0.749
OPG	0.071	0.207		0.067	0.251	0.037	0.508		0.024	0.673	-0.018	0.897		-0.092	0.506
t-PA	0.243	0.299		0.206	0.375	0.150	0.513		-0.070	0.759	-0.023	0.956		-0.242	0.550
TWEAK	-0.042	0.489		-0.045	0.478	0.006	0.923		-0.003	0.958	0.108	0.493		0.023	0.889
VEGF-A	-0.047	0.499		-0.063	0.355	0.005	0.942		-0.031	0.656	0.036	0.794		0.056	0.700
4E-BP1	-0.127	0.353		-0.152	0.263	0.107	0.401		0.060	0.652	0.314	0.238		0.227	0.422

^a^The Model 1 is adjusted for age

^b^The Model 2 model is adjusted for age, height, weight, body mass index, low density lipoprotein, triglycerides, systolic blood pressure, insulin, and C-reactive protein

## Discussion

In the present investigation, we have studied the association between the rs2043211 polymorphism in the *CARD8* gene and protein expression levels in a selection of 15 proteins involved in inflammation, evaluated by Olink proteomics in young healthy individuals. We showed that the polymorphism rs2043211 in the *CARD8* gene was significantly associated with lower levels of CCL20 and IL-6 in men. Both IL-6 and CCL20 have been associated with the atherosclerotic process. The chemokine CCL20 has been associated with overexpression in atherosclerotic lesions and is involved in the attraction of immune cells [23]. It signals via the Ccr6 receptor and the importance of the CCL20/Ccr6 axis for development of atherosclerosis was demonstrated in ApoE^−/−^ mice lacking Ccr6, which had reduced size of the atherosclerotic lesions [24]. The cytokine IL-6 has both pro- and anti-inflammatory properties [25]. It contributes to atherosclerosis via promotion of smooth muscle cell proliferation, migration and endothelial dysfunction [26]. In addition, IL-6 is involved in the production of acute phase proteins, has chemotactic abilities and can activate the endothelium, thereby facilitating the infiltration of immune cells into the atherosclerotic lesion [23]. On the other hand, IL-6 can also contribute to anti-inflammatory properties, such as inhibition of tumor necrosis factor (TNF)-α and IL-1 [25].

In the present study, the association between the polymorphism rs2043211 in the *CARD8* gene and reduced levels of CCL20 and IL-6 in men was significant both in the additive and dominant models, but not in the recessive model. Although the nature of the truncated variant of the *CARD8* gene is not clear, our data suggests that being heterozygous for the rs2043211 polymorphism in the *CARD8* gene is enough to alter the levels of specific proteins involved in inflammation. For CCL20 and IL-6, these results are consistent with our previous data [3], where we observed that knock down of *CARD8* in HUVECs generated a reduction of both CCL20 and IL-6 [3].

The remaining proteins showed inconsistent associations and/or no association in all three models, and this was inconsistent with our previous study [3]. However, in the additive model, we also observed that eight of the 15 proteins were altered in the same direction as in our previous study [3], although with non-significant associations.

We found an allele frequency of 69% for the major (T) allele and 31% for the minor (A) allele. This is in similar frequencies as described in the SNP database at National Center for Biotechnology Information (NCBI; https://www.ncbi.nlm.nih.gov/snp/rs2043211), where 68% of the European population carry the T allele and 32% the A allele. However, when stratifying the women by estrogen use, we observed a minor difference in the distribution of the alleles. Although smaller groups, the examination of women by estrogen use is a strength of our study, as it has been shown to be influential in other studies [18].

### Strengths

The data in the present study represent a unique contribution to the knowledge on CARD8, since very few studies have so far been published on the association between *CARD8* genotype and biomarkers of inflammation. The present study fills a gap in the research field via its preclinical approach by studying a young non-smoking healthy cohort. Although the focus of the LBA study is to discover biomarkers for early atherosclerosis, the results of the present study does not limit to CVDs, since it mirrors the general cytokine profile in young healthy individuals. Identification of markers of inflammation in healthy individuals may in the future contribute to decisions on early prevention management and thereby public policies related to health.

### Limitations

In the present study, we have utilized a cohort of young Swedish healthy non-smoking individuals to study the association between *CARD8* genotype and expression of chemokines and cytokines. However, the design of the present study does not permit conclusions on biomedical mechanistical causality between *CARD8* genotype and the expression of inflammatory proteins per se. Another potential limitation of the present study was that we could not statistically control for the physiological estrogen levels in the women participating in the study. Estrogen use increases the levels of CRP which has been observed both by others and by us [19, 20, 27]. In the present study we assumed that proteins involved in inflammation are also affected by estrogen use, like CRP. In general, most of the significant associations in the present study were observed in men. This may be a result of NEU and EU women being differentially influenced by either physiological cyclic variations and/or supplementary estrogen compared to men. However, we did not have data to adjust for cyclic variations, and the investigation of reasons of the differences in the associations by men and NEU and EU women is beyond the scope of the present study.

Another potential limitation of the present study is the generally low variance of inflammatory markers in the dataset. This is however expected in healthy young individuals.

In our previous publication [3], we showed that knock-down via siRNA of *CARD8* in human umbilical vein endothelial cells (HUVECs) resulted in lower expression of markers of inflammation. The comparability between our previous in vitro study made on HUVECs [3] and the present study in healthy young individuals is limited. Adjustments for risk factors on proteomics data based on in vitro cell culture were however not relevant in our previous investigation due to the nature of the study material and thereby not completely possible to compare. In the present study, we have adjusted for age (Model 1) and established risk factors for CVD (Model 2). However, with a few exceptions, adjustment according to Model 1 and Model 2 in the present study show similar results, implicating that the traditional risk factors play a limited role for the regulation of the levels of proteins involved in inflammation in relation to *CARD8* genotype.

### Future directions

Additional in vitro studies on the mechanistic effect of the truncated variant of CARD8 on inflammatory markers are warranted, especially under the influence of estrogen as a possible factor important for the inflammatory response. Another logical step will be to replicate the present study in an older and/or diseased study cohort.

## Conclusions

Collectively, in the present study using the LBA cohort which consists of healthy young adults, we have shown an association between the polymorphism rs2043211 encoding a truncated variant of the *CARD8* gene and lower levels of CCL20 and IL-6 levels in men. Our data indicate that CARD8 may be involved in the regulation of these proteins. No such associations were, however, evident for women, and the reason to this is still unclear.

### Supplementary Information

**Additional file 1: Supplementary material S1.** Distribution of proteins by TT, TA, AA, stratified by men, women with and without estrogen contraceptive use. **Supplementary material S2. **The script used in the study.

## Data Availability

Data sets generated and analyzed in the current study are not public available due to research subject confidentiality but are aware in a de-identified form from the corresponding author and PI upon reasonable request. The samples are being stored in Örebro Biobank no 454.

## Abbreviations

4E-BP1: Eukaryotic translation initiation factor 4E-binding protein 1
ADA: Adenosine deaminase
Apo: Apolipoprotein
AXL: Tyrosine-protein kinase receptor UFO
BMI: Body mass index
CAD: Coronary artery disease
CARD8: Caspase activation and recruitment domain 8
CCL20: C–C motif ligand 20
Ccr6: C–C motif chemokine receptor 6
CD: Cluster of differentiation
CRP: C-reactive protein
CVD: Cardiovascular disease
CXCL: (C-X-C motif) ligand
DNA: Deoxyribonucleic acid
EU: Estrogen users
FDR: False discovery rate
HEK: Human embryonic kidney
HUVEC: Human umbilical vein endothelial cells
IL: Interleukin
LBA: Lifestyle, biomarkers and atherosclerosis
LDL: Low density lipoproteins
LOD: Limit of detection
MCP: Monocyte Chemoattractant Protein
mRNA: Messenger Ribonucleic acid
NEU: Non-estrogen users
NF: Nuclear factor
NLRP3: NACHT, LRR and PYD domains-containing protein 3
OPG: Osteoprotegerin
PCR: Polymerase chain reaction
PDGF: Platelet-derived growth factor
SD: Standard deviation
SNP: Single nucleotide polymorphism
TNF: Tumor necrosis factor
t-PA: Tissue-type plasminogen activator
TWEAK: TNF-related weak inducer of apoptosis
VEGF: Vascular endothelial growth factor
