# Supplementary material for: Association between C10X polymorphism in the CARD8 gene and inflammatory markers in young healthy individuals in the LBA study
Source: BMC Cardiovasc Disord. 2024 Feb 13;24:103. doi: 10.1186/s12872-024-03765-7 (PMC10863129; doi:10.1186/s12872-024-03765-7)
Supplement: Supplementary file 1 — Additional file 1: Supplementary material S1. Distribution of proteins by TT, TA, AA, stratified by men, women with and without estrogen contraceptive use. Supplementary material S2. The script used in the study. [file 12872_2024_3765_MOESM1_ESM.docx]

**Supplementary material**

**Supplementary material S1. Distribution of proteins by TT, TA, AA, stratified by men, women with and without estrogen contraceptive use**

**Men**

**Women without estrogen contraceptive use (NEU women)**

**Women with estrogen contraceptive use (EU women)**

**Supplementary material S2. The script used in the study.**

# This script shows analyses for male

# Other groups were analysed by replacing the data

# Data were prepared by Stata

library(readstata13)

library(limma)

#*******#

# ADDTIVE MODEL, AGE-ADJUSTED

#*******#

rm(list = ls())

dat <- read.dta13(".../male.dta")

View(dat)

design_adj <- model.matrix(~tttaaa

+ dat$agesp1 + dat$agesp2 + dat$agesp3

, data=dat)

fit_adj <- lmFit(t(dat[45:ncol(dat)]), design=design_adj)

fit_adj

ebays_fit_adj<-eBayes(fit_adj)

DE_results_adj <- topTable(ebays_fit_adj,coef="tttaaa", adjust.method="fdr", confint=TRUE, n=Inf)

DE_results_adj

#*******#

# ADDTIVE MODEL, MOST ADDTIVE MODEL, ADJUSTED

#*******#

rm(list = ls())

dat <- read.dta13(".../male.dta")

design_adj <- model.matrix(~tttaaa

+ dat$agesp1 + dat$agesp2 + dat$agesp3

+ dat$bmisp1 + dat$bmisp2 + dat$bmisp3

+ dat$ldlsp1 + dat$ldlsp2 + dat$ldlsp3

+ dat$triglysp1 + dat$triglysp2 + dat$triglysp3

+ dat$insulinsp1 + dat$insulinsp2 + dat$insulinsp3

+ dat$systolicbp_1sp1 + dat$systolicbp_1sp2 + dat$systolicbp_1sp3

, data=dat)

fit_adj <- lmFit(t(dat[45:ncol(dat)]), design=design_adj)

fit_adj

ebays_fit_adj<-eBayes(fit_adj)

DE_results_adj <- topTable(ebays_fit_adj,coef="tttaaa", adjust.method="fdr", confint=TRUE, n=Inf)

DE_results_adj

#*******#

# DOMINANT OR RECESSIVE MODEL, AGE-ADJUSTED

#*******#

rm(list = ls())

dat <- read.dta13(".../male.dta")

run_limma <- function(groups) {

dataset_group <- dat[dat$dominant %in% groups, ]

print(table(dataset_group$dominant))

design <- model.matrix(~0 + as.factor(dataset_group$dominant)

+ dataset_group$agesp1 + dataset_group$agesp2 + dataset_group$agesp3)

colnames(design) <-c("control", "case", "agesp1", "agesp2", "agesp3")

contrast <- makeContrasts(Diff = case-control, levels=design)

print(head(contrast))

fit <- lmFit(t(dataset_group[45:ncol(dataset_group)]), design=design, maxit=1000)

contrast_fit<-contrasts.fit(fit,contrast)

ebays_fit<-eBayes(contrast_fit)

print(summary(decideTests(ebays_fit)))

DE_results <- topTable(ebays_fit,n=ncol(dataset_group), adjust.method="fdr", confint=TRUE)

return(DE_results)

}

group_0_vs_1 <- run_limma(groups=c(0,1))

group_0_vs_1

#*******#

# DOMINANT OR RECESSIVE MODEL, MOST ADJUSTED

#*******#

rm(list = ls())

dat <- read.dta13(".../male.dta")

run_limma <- function(groups) {

dataset_group <- dat[dat$dominant %in% groups, ]

print(table(dataset_group$dominant))

design <- model.matrix(~0 + as.factor(dataset_group$dominant)

+ dataset_group$agesp1 + dataset_group$agesp2 + dataset_group$agesp3

+ dataset_group$bmisp1 + dataset_group$bmisp2 + dataset_group$bmisp3

+ dataset_group$ldlsp1 + dataset_group$ldlsp2 + dataset_group$ldlsp3

+ dataset_group$triglysp1 + dataset_group$triglysp2 + dataset_group$triglysp3

+ dataset_group$insulin2sp1 + dataset_group$insulin2sp2

+ dataset_group$systolicbp_1sp1 + dataset_group$systolicbp_1sp2 + dataset_group$systolicbp_1sp3)

colnames(design) <-c("control", "case", "agesp1", "agesp2", "agesp3", "bmisp1", "bmisp2", "bmisp3", "ldlsp1", "ldlsp2", "ldlsp3", "triglysp1","tryglysp2", "tryglysp3", "insulin2sp1", "insulin2sp2", "systolicbp_1sp1", "systolicbp_1sp2","systolicbp_1sp3")

contrast <- makeContrasts(Diff = case-control, levels=design)

print(head(contrast))

fit <- lmFit(t(dataset_group[45:ncol(dataset_group)]), design=design, maxit=1000)

contrast_fit<-contrasts.fit(fit,contrast)

ebays_fit<-eBayes(contrast_fit)

print(summary(decideTests(ebays_fit)))

DE_results <- topTable(ebays_fit,n=ncol(dataset_group), adjust.method="fdr", confint=TRUE)

return(DE_results)

}

group_0_vs_1 <- run_limma(groups=c(0,1))

group_0_vs_1
